# Pregnancy vulnerability in urban areas: a pragmatic approach combining behavioral, medico-obstetrical, socio-economic and environmental factors

**DOI:** 10.1038/s41598-019-55005-0

**Published:** 2019-12-11

**Authors:** Alice Brembilla, Nadine Bernard, Sophie Pujol, Anne-Laure Parmentier, Astrid Eckman, Anne-Sophie Mariet, Hélène Houot, Quentin Tenailleau, Gérard Thiriez, Didier Riethmuller, Marie Barba-Vasseur, Frédéric Mauny

**Affiliations:** 1 UMR 6249, Laboratoire Chrono-environnement, Université de Bourgogne Franche-Comté, CNRS, F-25000 Besançon, France; 2 Unité de méthodologie en recherche clinique, épidémiologie et santé publique (uMETh), Inserm CIC 1431, CHU, Besançon, France; 3 Centre National de la Recherche Scientifique, UMR 6049, Laboratoire ThéMA, Université de Bourgogne Franche-Comté, Besançon, France; 4 Service de Gynécologie-Obstétrique, CHU, F-25000 Besançon, France; 5 Service de biostatistique et d’informatique médicale (DIM), Centre Hospitalier Universitaire, Dijon, France; 6 Laboratoire LADYSS, UMR 7533, Université Paris Ouest - Nanterre - la Défense, Nanterre, France; 7 Service de Réanimation Pédiatrique, Néonatalogie et Urgences Pédiatriques, CHU, F-20500 Besançon, France; 8 Present Address: EA 4483, Laboratoire sciences végétales et fongiques, Université de Lille, Lille, France

**Keywords:** Epidemiology, Environmental impact

## Abstract

Multiple risk factors are associated with adverse pregnancy outcomes (APO), but how all these different factors combine and accumulate remains unknown. The objective of this observational retrospective study was to describe the accumulation of multiple vulnerability markers in pregnant women living in an urban area. Women living in Besançon (France) who delivered between 2005 and 2009 were included. Individual data were collected from the obstetrical records while environmental exposures were collected using environmental prediction models. The accumulation of 15 vulnerability markers, grouped into six dimensions (maternal age, smoking, body mass index (BMI), socio-economic, medico-obstetrical and environmental vulnerabilities) was described and analyzed in comparison with four APO. Among the 3686 included women, 20.8% were aged under 20 or over 34 and 21.9% had an extreme pre-pregnancy BMI. 18.8% declared smoking during pregnancy. Women exposed to socio-economic, medico-obstetrical or environmental vulnerability were 14.2%, 31.6% and 42.4% respectively. While 20.6% were not exposed to any marker, 18.8% accumulated three or more dimensions. The risk of APO increased significantly with the cumulative number of vulnerabilities. Define and validate a vulnerability score could be useful to identify vulnerable women, adapt their pregnancy monitoring and help policy makers to implement appropriate education or health promotion programs.

## Introduction

Adverse pregnancy outcomes (APO), such as preterm birth (PTB), low birthweight (LBW) preeclampsia or ante-partum hemorrhage, are important indicators for the health of the newborns and infants. In addition, growth and developmental delays in utero may influence the subsequent health status of individuals, including increased mortality and morbidity in childhood and an elevated risk of coronary heart disease, hypertension and diabetes in adulthood^1,2^. Multiple and varied etiologies and/or risk factors are associated with these APO; they combine in complex ways and could be related to medico-obstetrical, socio-demographic, behavioral and/or environmental determinants.

Obstetric history is well known to influence pregnancy outcome. Women with history of prior PTB have indeed higher risk for preterm delivery in a subsequent pregnancy compared with those with a prior term birth^3^. Abnormalities of the female reproductive tract and uterine scar also increase the risk for adverse pregnancy outcome^4,5^. Various pregnancy characteristics have been associated with PTB, LBW, preeclampsia or ante-partum hemorrhage. Obstetrical disorders such as hypertension, diabetes, genitourinary infections are identified as risk factors of adverse birth outcomes^6^. Multiple gestations carry a substantial risk of preterm delivery, and results in 15–20% of all PTBs. Nearly 60% of twins are born preterm^6^. Of course, multiple gestations are also closely linked to birthweight (BW). After 33 weeks of gestation, BW of twins started to deviate from singletons (difference of 900 grams at 42 weeks)^7^.

Demographic variables such as extremes in maternal age or black maternal ethnicity are also recognized as associated with APO^8,9^. The extremes of maternal pre-pregnancy body mass index (BMI) seem to be linked with an increase of the overall incidence of APO^10^. According to a recent meta-analysis, pre-pregnancy underweight increases the risk of LBW^11^. Some maternal behaviors are identified as preventable risk factors for an unsuccessful pregnancy outcome. Smoking during pregnancy has been associated with fetal growth restriction, placenta praevia (PP) and PTB^12,13^. Weaker evidence suggests an adverse effect of environmental tobacco smoke, heavy alcohol or cocaine use^14^. Socio-economic deprivation has been shown to be linked to access to care, behaviors during pregnancy, and pregnancy outcomes^15–17^.

The last decade provide an increasing evidence of the link between environment and pregnancy outcome. A higher risk of fetal growth restriction in relation to air pollution exposure was reported by main of the studies dealing with carbon monoxide (CO), nitrogen dioxide (NO_2_) or particulate matter (PM_10_, PM_2.5_)^18–21^. Effects on PTB appears to be more discussed^18–20,22^. According to a recent review of the literature, there is some suggestive evidence of adverse associations with environmental noise, especially for LBW^23,24^. Otherwise, few studies demonstrate a benefic effect of maternal proximity to green spaces on BW^25,26^.

However, most studies focus on only one determinant or one class of determinant and/or their influence on pregnancy outcome (when considered, the other determinants are treated as potential confounding variables). So, the distribution of all these determinants and how they potentially accumulate in the population of pregnant women is still largely unknown. Considering simultaneously all of these determinants could help define a concept of global vulnerability. This concept of vulnerability is very broad here: it is not only a socio-economic vulnerability but an interaction between various causes: genetic, socio-economic, psychological and environmental… To our knowledge, such a descriptive approach was never conducted in a large pregnant women population-based study.

The main objective of this article was to describe the distribution of identified vulnerability markers related to medico-obstetrical, demographic, behavioral and environmental dimensions, and to describe their potential accumulation in a population of pregnant women living in a middle-sized urban area (Besançon, France). Indeed, in Europe, cities of 100,000 to 500,000 inhabitants are considered to be “medium-sized”^27^. They define the largest category of city in demographic terms, hosting more than 44% of the European population^28^.

The second objective was to analyze the relationship between vulnerability accumulation and some adverse pregnancy complications and outcomes: preeclampsia, vaginal bleeding in the second and third trimesters, PTB and LBW.

## Methods

### Population

This epidemiological observational retrospective study included all pregnancies resulting in singleton births that occurred in the University Hospital of Besançon between 1 January 2005 and 31 December 2009 and whose mother, at the delivery, aged 18 and over and lived in Besançon City (a medium-sized city in France). Both stillborn and liveborn infants newborns whose birth occurred after 22 completed weeks of gestation and/or with a birthweight of 500 g or above were included. Mothers were considered only once in the study to ensure independence of observations: in case of repeated deliveries during the period, one episode has been randomly selected and the others were excluded. Multiple pregnancies were not included because of special characteristics in terms of duration and fetal growth compared to all pregnancies. Induced abortions, pregnancies with missing or invalid data for delivery date or address of residence at the baby’s birth were excluded.

### Study variables

All except environmental markers were obtained from the computerized obstetrical record of Besançon University Hospital (DIAMM^TM^ software), using International Classification diseases ICD-10 codes which were inserted by clinicians at the date of completion of the medical records.

#### Vulnerability variables

A pragmatic approach was conducted to select variables that could be used to define vulnerability. Selection criteria were as follows. To be retained, factors should be suspected or identified as risk factors of adverse birth outcomes. They should concern the period before pregnancy or the first trimester of pregnancy, which was defined by a window period of 15 weeks from the date of last menstrual period. Among them, medico-obstetrical and demographic markers were retained according to the data availability in the personal medico-obstetrical records, and were collected during current management of delivery. Furthermore, accuracy in the medical database had to be very high, and missing data had to be very low. For example, due to the retrospective nature of the study, known factors of APO such as alcohol consumption during pregnancy had to be excluded from the study because of important downward bias. The women address at the date of delivery was used to geocode the residential building and to define characteristics of the environmental living neighborhood. Fifteen variables were finally retained to describe pregnancy vulnerability (Table 1).

**Table 1:** Variables used to define the pregnancy vulnerability markers.

Variables	Definition
Extreme maternal age	Maternal age at delivery <21 years old or ≥35 years old (yes/no)
Pre-pregnancy body mass index	Pre-pregnancy underweight (BMI < 18.5 kg/m^2^) or pre-pregnancy obesity (BMI ≥ 30 kg/m^2^) computed with the BMI collected at the first antenatal visit
Maternal smoking	Declared maternal smoking during pregnancy (yes/no)
*Medico-obstetrical markers*	Previous preterm birth referred to a history of any PTB < 37^+0^ weeks
	Uterine scare or abnormalities of the female reproductive tract referred to codes O34 and Q50 to Q52 (ICD - 10th version)^a^
	Hypertension: chronic pre-pregnancy blood pressure >140/90 mmHg and for all hypertension disease diagnosed during pregnancy, excluding eclampsia and preeclampsia (ICD-10 codes: I10 to I15, O10, O11, O13, O16 and P000).
	Diabetes: gestational diabetes or a history of diabetes (ICD-10 codes: E10 to E14, H360, N083, O24, P700 and P701).
	Infection of genitourinary tract in pregnancy: ICD-10 codes: O23, A181, A510, [A540; A542], [A560; A562], A590 and A600.
	Assisted reproductive technology use (yes/no)
Socio-economic markers	Mother living alone (yes/no)
	Maternal professional status in current pregnancy: “unemployment” (yes/no)
*Environment and neighborhood*	Women living in an IRIS ranked in the tenth decile SES (yes/no)
	Air pollution (average level of NO_2_ ≥ 40 µg/m^3^ in front of residential building during at least one month of pregnancy) (yes/no)
	Noise: night noise average in front of residential building (L_Aeq_ > 55 dB) (yes/no)
	Lack of wooded area in the 100 meters around the residence building (yes/no)

^a^ICD-10: International Statistical Classification of Diseases and Related Health Problems of the WHO, 10^th^ edition.

The neighborhood deprivation index was created for the city of Besançon, according to the approach developed by Lalloué *et al*.^29^. The statistical unit was the IRIS (Îlots Regroupés pour l’Information Statistique), a geographical unit currently used by the French National Institute of Statistics and Economic Studies for population censuses (approximately 2000 individuals with relatively homogeneous social characteristics). Variables related to family and household, immigration and mobility, employment and income, education and housing were extracted from 2008 INSEE database. Eighteen variables were selected among the 39 variables most often used in the literature^29–31^. (Supporting Information). The first component of a principal component analysis (PCA) was used to calculate the socioeconomic index after a reduction step and standardization. Women living in an IRIS ranked in the tenth decile of the socio economic index were considered to have a very low neighborhood deprivation level.

Noise and NO_2_ environmental exposures have been assessed by environmental prediction models. The same inputs were used for the two models: meteorological observations, topographic data, shape, size, height and position of both roads and buildings, railway and road traffic data for each segment of the city^32^. Maps of night noise levels and NO_2_ levels have been modeled at the city scale, using the MITHRA-SIG (noise), Circul’Air and ADMS-Urban (air) softwares^21,22,33,34^. Both noise and NO_2_ maps have been validated by measurement campaigns. To account for the temporal variability of weather conditions and the seasonal variations in concentrations of pollutants in the air, monthly maps have been used to calculate indicators of exposure to NO_2_. For each woman, monthly NO_2_ exposure level during pregnancy has been calculated. European NO_2_ threshold of 40 µg/m^3^ and the WHO threshold of 55 dB(A) for the night noise level (L_Aeq,night_) were used to define environmental exposure^35^.

Vulnerability was considered in two steps. The 15 markers were first independently analyzed. Then, 6 vulnerability dimensions were created (and coded as at least one marker observed: yes or no). Three dimensions were single-marker (“extreme” maternal age, maternal smoking during pregnancy, pre-pregnancy body mass index) and three dimensions were multi-markers (medico-obstetrical, socio-economic status, environment and neighborhood). Because it could not be excluded that hypertension, diabetes and/or genitourinary infection markers records could concern events that had occurred during second or third trimester of pregnancy, sensitivity analyses were performed by successively removing these markers.

#### Adverse pregnancy outcomes

Gestational age at delivery was based on the last menstrual period or on ultrasonography during the first trimester. Preterm delivery was defined by a childbirth before 37 weeks of pregnancy and LBW by a weight at birth under 2500 grams. Vaginal bleeding in the second and third trimesters referred to an episode of bleeding after 28 weeks of gestation, including retro-placental hematoma and bleeding because of placenta praevia (International Classification diseases ICD-10 codes: O45, O46, O441 and P021). Preeclampsia and eclampsia referred to ICD-10 codes O14 and O15.

### Statistical analysis

The results of the descriptive phase are expressed as frequencies and percentages (%) and 95% confidence interval (CI). Chi-square test was used to test for the difference of distribution of vulnerability among the pregnant women with or without the medical events. Cochran-Armitage test was used to test for trend of increasing or decreasing of the medical events in each of the vulnerability classes. Significance level was set at P < 0.05. Bivariate logistic regressions were performed to analyze the relationship between each APO and vulnerabilities or dimensions classes. Predicted probabilities of APO were calculated for each combination of the six dimensions.

Databases were managed with SAS version 9.4 software (SAS Institute, Cary, NC) and Microsoft Excel 2010. Statistical analyses were performed with R and SAS 9.4, the R package SesIndexCreatoR^29^ for the creation of neighborhood deprivation index and R package ggplot2 for plots.

### Ethical approval and informed consent

This study was approved by the French National Advisory Committee for the Treatment of Information in Health Research (CCTIRS) and by the French data protection authority (CNIL) [Reference number: 915261]. All methods were carried out in accordance with the ethical standards of CNIL and the Declaration of Helsinki. The requirement for patient consent was waived by the CNIL because of the retrospective nature of the study. A letter of information was sent to each participant. Women who opposed the processing of their data were excluded from the study. All records were anonymized prior to analysis.

## Results

Among the 11 630 births identified, 4622 births (39.7%) with mother’s home address located in Besançon. Among them, 936 births were excluded for the following reasons: Families’ objection to processing of their medical data (n = 10), maternal age less than 18 years (n = 37), missing delivery date (n = 3), missing birthweight (n = 2), birthweight under 500 grams (n = 4), unidentifiable address of residence (n = 14), multiple pregnancies (n = 199) and excluded episodes due to repeated pregnancies (n = 667). Finally, 3686 singleton pregnancies were included in the analysis.

### Characteristics of mothers, pregnancies and newborns

The observed maternal age was 29.6 years (standard deviation SD = 5.5) on average. Over half the number of mothers were nulliparous and 67% delivered by non-instrumental vaginal delivery. The average birthweight was 3225.0 g (SD = 573.4). The prematurity rate was 7.2% and the rate of low birthweight was 7.3% (Table 2).

**Table 2:** Characteristics of mothers, pregnancies and newborns included in the study (N = 3686).

	Total number	%
**Maternal age (years)**		
<20	119	3.2
20–24	668	18.1
25–29	1229	33.3
30–34	1023	27.8
35–39	504	13.7
> = 40	143	3.9
**Parity**		
0	1960	53.2
1	979	26.6
2	447	12.1
> = 3	300	8.1
Preeclampsia or eclampsia	103	2.8
Vaginal bleeding (2nd or 3th trimester)	105	2.8
**Mode of delivery**		
Vaginal	2459	66.7
Instrumental extraction	729	19.8
Caesarean	498	13.5
**Gestational age at delivery (weeks of amenorrhea)**		
<28	20	0.5
28–31	45	1.2
32–36	202	5.5
> = 37	3419	92.8
Sex^a^		
Male	1926	52.3
Female	1756	47.7
**Apgar score at five minutes**		
< = 7	61	1.7
8–9	127	3.4
10	3498	94.9
Birthweight (grams) [mean(SD)]	3225.0	(573.4)
Low birthweight^b^	269	7.3

^a^Missing data: sex (n = 4).

^b^Weight at birth less than 2500 grams.

### Pregnancy vulnerability

The distributions of each vulnerability marker alone and among the 6 dimensions are presented in Table 3.

**Table 3:** Pregnancy vulnerability distribution (N = 3,686): Marker alone and grouped in six dimensions.

	n	% [95% CI]
Maternal age <20 years or ≥35 years	766	20.8 [19.5–22.1]
Maternal smoking during pregnancy	694	18.8 [17.6–20.1]
Pre-pregnancy body mass index <18.5 or ≥30	806	21.9 [20.5–23.2]
Medico-obstetrical markers		
Uterine scare or abnormality of the female reproductive tract	209	5.7 [4.9–6.5]
Previous preterm birth	52	1.4 [1.1–1.8]
Hypertension^a^	172	4.7 [4.0–5.4]
Diabetes^b^	217	5.9 [5.1–6.7]
Infection of genitourinary tract in pregnancy	636	17.3 [16.0–18.5]
Assisted reproductive technology use	73	2.0 [1.6–2.5]
*At least one vulnerability marker of this dimension*	1163	31.6 [30.1–33.1]
Socio-economic markers		
Mother living alone	337	9.1 [8.2–10.1]
Maternal unemployment during pregnancy^c^	221	6.0 [5.3–6.8]
*At least one vulnerability marker of this dimension*	524	14.2 [13.1–15.4]
Environmental and neighborhood markers		
Neighborhood deprivation index: tenth decile	594	16.1 [14.9–17.3]
Noise >55 dB LAeq,night^d^	775	21.0 [19.7–22.4]
NO2 ≥ 40 μg/m^3e^	138	3.7 [3.2–4.4]
Lack of wooded area in the 100 meters around the residence building	520	14.1 [13.0–15.3]
*At least one vulnerability marker of this dimension*	1562	42.4 [40.8–44.0]

^a^History of hypertension or hypertensive disease of pregnancy, excluding preeclampsia and eclampsia.

^b^History of diabetes or gestational diabetes.

^c^Does not include housewives.

^d^Noise average in front of residential building >55 dB LAeq,night (sound pressure level in dB, equivalent to the total sound energy over the night period 22h-6h).

^e^Average level of NO2 ≥ 40 μg/m3 during at least one month of pregnancy.

The proportion of women aged under 20 or over 34 years old at the baby’s birth was 20.8%, while 21.9% of women had a pre-pregnancy BMI < 18.5 or ≥30 kg/m^2^. Maternal smoking during pregnancy was declared by 18.8% of included women. Fourteen percent of the woman presented one or more markers of socio economic vulnerability; this percent reached 31.6 and 42.4 when considering one or more medico-obstetrical and environmental vulnerabilities, respectively.

### Distribution of women according to their vulnerabilities accumulation

**Figure 1:**
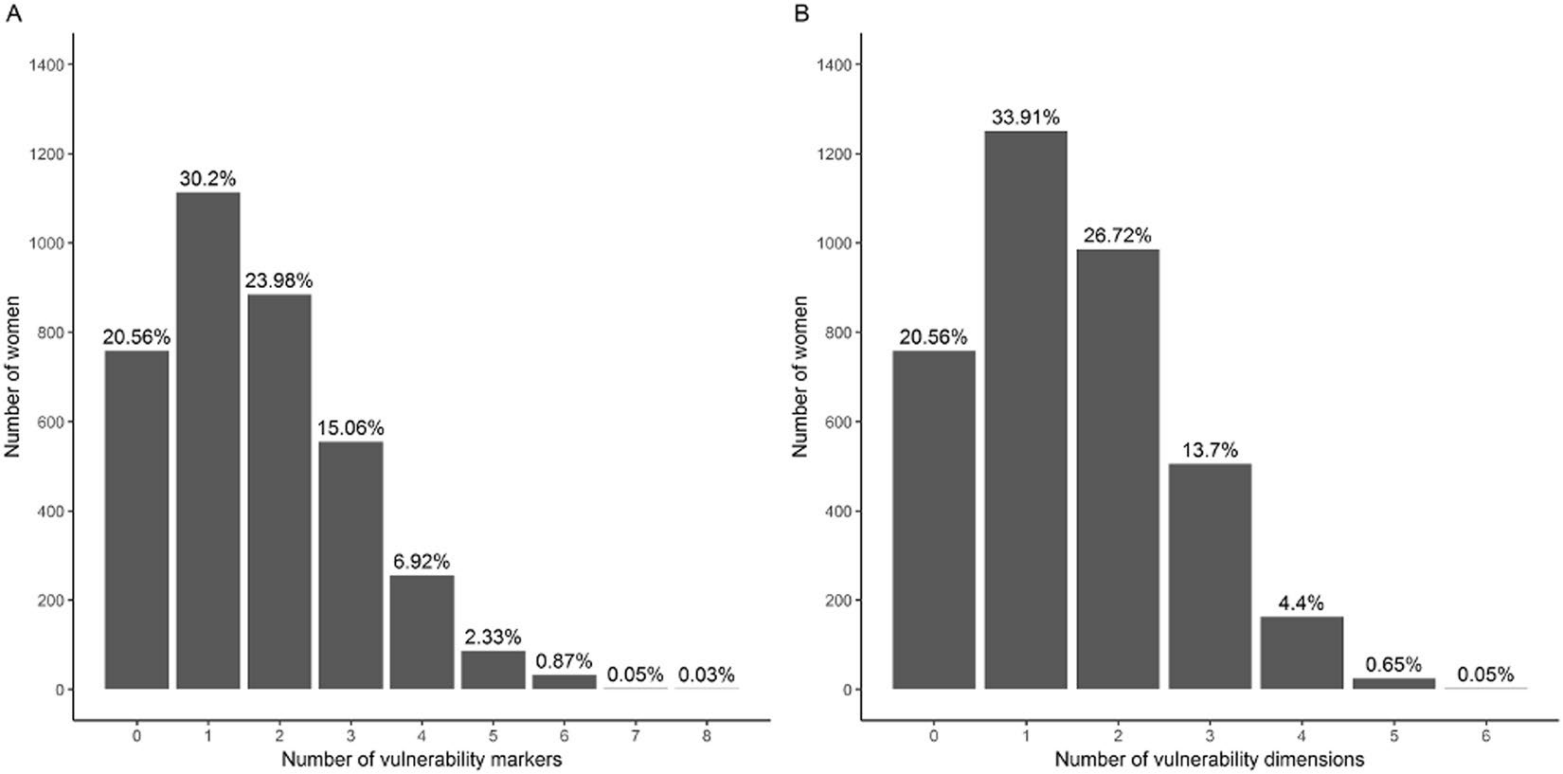
Distribution of women according to their cumulative exposure to the 15 vulnerability markers (**A**) and to the 6 vulnerability dimensions (**B**).

No marker of vulnerability was recorded for 758 women (20.6%), while four markers or more were simultaneously recorded for 376 women (10.2%) (Fig. 1A); 188 (5.1%) women were concerned by four or more of the six vulnerability dimensions (Fig. 1B). One woman was exposed to eight of the fifteen vulnerability markers and two women to the six vulnerability dimensions.

### Vulnerabilities accumulation and proportions of APO

An increase of the percent of APO was observed when the number of vulnerability markers increased; the same increasing trends were observed when considering the vulnerability dimensions (all P ≤ 0.03) (Supplementary Tables S1 and S2). Tables 4 and 5 summarize the odds-ratio (OR) of APO according to the observed number of vulnerability markers (Table 4) or the observed number of vulnerability dimensions (Table 5). APO were associated with a higher number of vulnerabilities and dimensions. For example, a higher number of dimensions was significantly associated with preterm birth, with OR = 1.49 (95% CI = 1.01–2.19 among women with two vulnerability dimensions and OR = 2.14 (95% CI = 1.24–3.72) among those with four or more of the six vulnerability dimensions, compared to women without any vulnerability (P = 0.037). Sensitivity analyses were performed by successively removing three markers: hypertension, diabetes and genitourinary infections. The results were very close to those observed with the 15 markers (Supplementary Tables S3 and S4).

**Table 4:** Vulnerability markers and odds-ratio of adverse pregnancy outcomes (N = 3686).

	At least one pregnancy outcome OR (95% CI, P)	Preterm birth	Low Birthweight	Vaginal bleeding	Preeclampsia-Eclampsia
Number of markers	P < 0.001	P = 0.011	P < 0.001	P = 0.011	P < 0.001
0	1	1	1	1	1
1	1.41 (1.02–1.94)	1.21 (0.82–1.79)	1.45 (0.96–2.19)	1.37 (0.68–2.76)	2.92 (0.98–8.72)
2	1.81 (1.31–2.50)	1.24 (0.83–1.86)	1.75 (1.15–2.66)	2.56 (1.32–4.97)	6.85 (2.41–19.49)
3	2.29 (1.63–3.22)	1.73 (1.13–2.64)	2.09 (1.34–3.26)	2.20 (1.06–4.58)	7.05 (2.40–20.72)
≥4	3.08 (2.16–4.41)	2.03 (1.29–3.19)	2.53 (1.58–4.04)	2.58 (1.20–5.58)	16.94 (5.94–48.33)

**Table 5:** Vulnerability dimensions and odds-ratio of adverse pregnancy outcomes (N = 3686).

	At least one pregnancy outcome OR (95% CI)	Preterm birth	Low Birthweight	Vaginal bleeding	Preeclampsia-Eclampsia
Number of dimensions	P < 0.001	P = 0.037	P < 0.001	P = 0.012	P < 0.001
0	1	1	1	1	1
1	1.47 (1.08–2.01)	1.20 (0.82–1.76)	1.45 (0.97–2.18)	1.53 (0.78–3.00)	3.69 (1.28–10.67)
2	1.97 (1.44–2.69)	1.49 (1.01–2.19)	1.88 (1.25–2.82)	2.43 (1.26–4.69)	7.15 (2.54–20.17)
3	2.50 (1.77–3.53)	1.59 (1.02–2.47)	2.27 (1.45–3.55)	2.83 (1.39–5.77)	8.99 (3.09–26.15)
≥4	2.84 (1.83–4.42)	2.14 (1.24–3.72)	2.46 (1.38–4.37)	1.35 (0.43–4.24)	17.53 (5.79–53.08)

**Figure 2:**
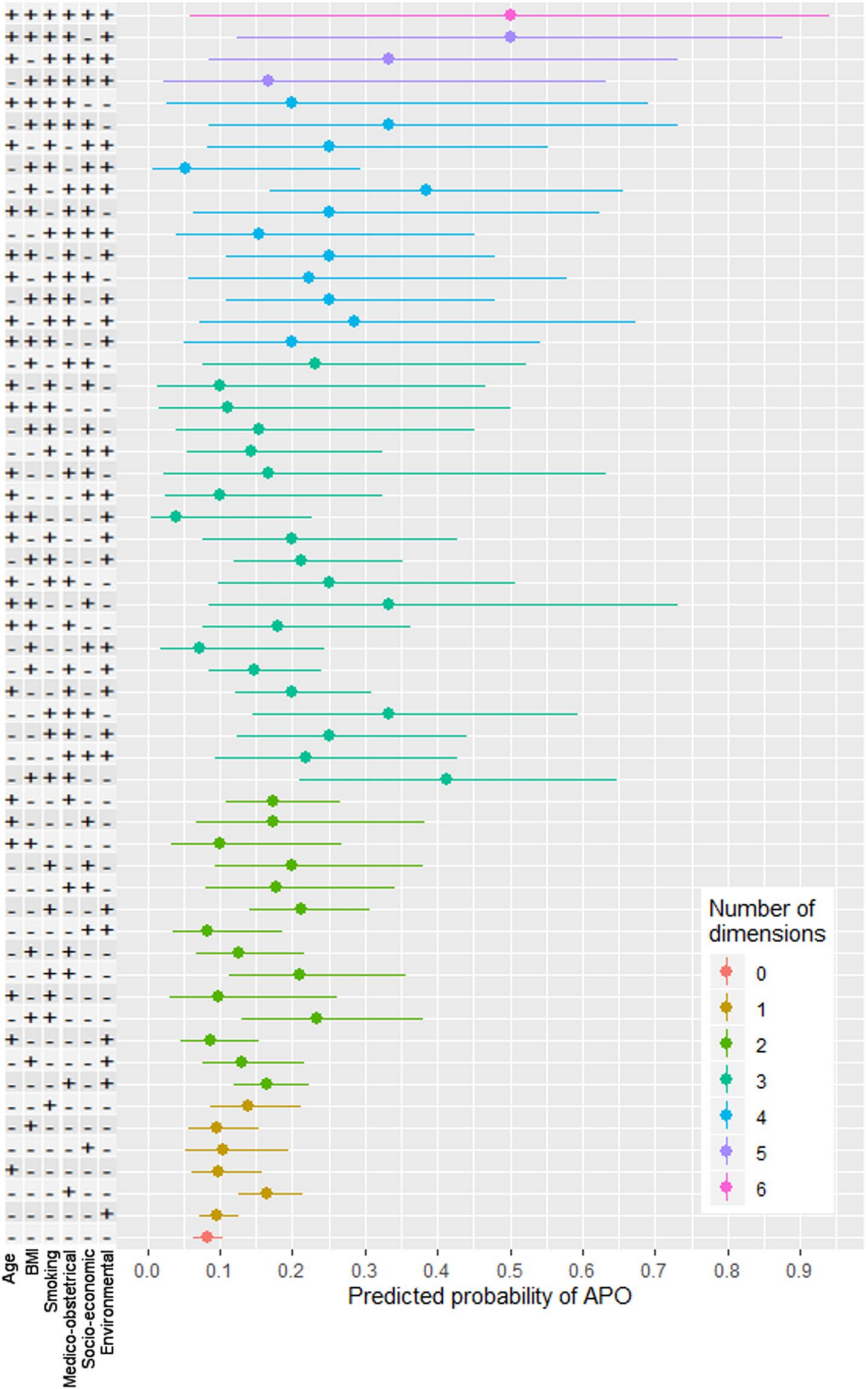
Predicted probabilities of at least one APO according to each combination of the 6 dimensions.

The modulation of the predicted probability of at least one APO among all the different combinations of the vulnerability dimensions is presented in Fig. 2.

## Discussion

The vulnerability markers were differently distributed among pregnant women, from less than 2% to more than 20%. However, about 80% of the pregnant women were concerned by at least one vulnerability marker and nearly half (45.5%) accumulated two or more markers. Eleven percent were exposed to at least four of the six vulnerability categories. In addition, the more women combined vulnerabilities, the more their probability of adverse birth outcomes increased.

The study population included patients from public university hospital and did not cover deliveries managed by the private hospital. However, the main characteristics observed in our sample were similar to those observed in the French perinatal survey performed in 2010, especially the mean of maternal age, the percentages of low birthweight and preterm birth^36^. The main limitation relates to the retrospective recording of vulnerability markers, especially the characterization of medico-obstetrical vulnerabilities and their distinction from APO. The retrospective collect of data from computerized obstetric records could have lead, in some cases, to misclassifications due to a lack of precision. But, when omitting the three less reliable markers, sensitivity analyses produced very close results. Several markers should have also been underestimated, especially smoking during pregnancy. Environmental markers were directly extracted from geocoded mother address using a completely reproducible process and a special attention was paid on collecting the actual address of women at delivery and not the last address recorded in the hospital information system.

The choice of the vulnerability markers was not easy and several factors could miss in the list. The selection was based on the literature and also on the availability of reliable data from medical records. Markers such as addictive behavior were not retained because of the risk of very low rate of identification. Despite being known as a major factor, ethnicity was not available for this approach. Because of legal restrictions, this information is not commonly recorded in official French databases. To select medical markers, we referred to the recommendations of the French National Authority for Health (HAS) about “monitoring and guidance of pregnant women based on risk situations identified”^37^. The goal was here to separate the diseases identified at the start of the pregnancy (or considered as vulnerabilities implying monitoring to prevent obstetrical complications) and the real APO (i.e. serious complications of late pregnancy, preterm birth or low birthweight). A history of hypertension or pregnancy-induced hypertension was then considered as medico-obstetrical risk factors while preeclampsia and eclampsia were considered as APO.

Environment markers were selected using the same rules. Road traffic is often the main source of air pollution in medium-sized cities like Besançon, and NO_2_ is a gaseous pollutant known to be a good indicator of road traffic^33^. Adverse effects are frequently encountered in population expose to noise: annoyance, disturbed sleep, increased risk of cardiovascular disease^38^. Thus, our team recently showed that these two former pollutants only partially overlap^32^. The presence or absence of wooded area in the 100 meters around the residence building were used as an indicator of surrounding greenness^25,26^. If some marker could be discussed, the similar results obtained by considering individually each marker or grouped by dimensions suggest that the results could not be too sensitive to the choice of the study markers.

To our knowledge, this is the first study that describes the exposure to multiple sources of vulnerability (both individual and environmental) during pregnancy in an urban area, without focusing on the effect of one factor or one class of factors on pregnancy outcome. Despite low to moderate proportion of women concerned by each of the selected markers, nearly 80% were exposed to vulnerability markers during pregnancy. Women with no vulnerability markers could be seen as slightly over-represented: considering the observed proportion of women among whom each marker was recorded, the expected proportion would be 16% (based on the hypothesis of independency of markers). Conversely, about 10% of women cumulated more than 3 vulnerability markers. Moreover, when considering the multi-markers dimensions, the gap between the “at least one marker” proportion and the proportion of each of the concerned markers suggest that the markers only partially overlap. Thus, our results suggest: i) a tendency for accumulation of markers in a part of the pregnant women, ii) an accumulation of markers from different vulnerability dimensions rather than from the same dimensions.

The dose/effect relationship between the number of the vulnerabilities the women accumulated and the probability of adverse birth outcomes enhance the interest of our approach. A partition of women in three groups could be proposed. Women with no marker are associated with a lower (but not null) probability of adverse pregnancy outcome. This is in line with the unexplained proportion of APO regularly observed, such as with PTB^39^. No threshold was identified, but women with two or more markers appeared to be over the national risk of adverse pregnancy outcome^36^. Finally, in an analytic point of view, markers are missing, such as genetic, behavioral and occupational factors, but our approach was pragmatic and focused on easily and currently recorded data.

One of the limits of the results relies on study site. Besançon city is a European “medium-sized” city (i.e. city of 100,000 to 500,000 inhabitants)^27^. Environmental pollution (especially air pollution) and social disparity are expected to be higher in larger cities, which could conduct to a higher proportion of accumulation of vulnerabilities markers in a part of the population living in deprived areas.

We worked on markers and dimensions approaches. In a clinical perspective, using only six dimensions would be easily useable and understandable, and a score would potentially be easier to use with only six dimensions, rather than fifteen markers. About the determination of relevant threshold on the number of markers or dimensions, the answer is not obvious as the first results seem to acknowledge a linear or monotonous growth depending on the markers’ accumulation, and needs to be determined. The results of our study are however not directly applicable, and will need to be confirmed with complementary analyses: Building a score, pondering the markers and then providing an external validation of this score. Modelling could also simultaneously take account of the different defined outcomes, using multivariate generalized linear mixed models, rather than using a unique restrictive “at least one” outcome. Concerning the environmental dimension, results are not currently directly applicable in routine practice. Indeed, environmental and neighborhood indicators are not automatically accessible for every patient. A solution could be the use of proxy indicators. For instance, the distance to a major pollution source such as railway or high traffic road, predefined neighborhoods classifications or small area contextual estimations. Maps with an integrated tool of query processing using home address could also be implemented in clinical structures for this purpose. Considering the growing interest for environmental impact of health, and the spreading awareness of environmental risks on maternal health, it is likely that such approaches will be increasingly proposed in routine practice in the foreseeable future.

In conclusion, by combining medical, behavioral, socio-economic and environmental markers, this pragmatic study shows that vulnerabilities are not homogenously distributed and tend to accumulate in a part of the pregnant women. This accumulation could be seen as a particular risk of adverse pregnancy outcome and could be connected to the concept of Exposome^40^. One perspective could be to define and validate a vulnerability score. This study confirms also the need to identify vulnerable women as early as possible during pregnancy to adapt their pregnancy monitoring. Moreover, targeting vulnerable populations of women across small local urban area level could help policy makers to implement appropriate education or health promotion programs to specific areas of the city.

## Supplementary information

Supplementary materials
