# Supplementary material for: Pregnancy vulnerability in urban areas: a pragmatic approach combining behavioral, medico-obstetrical, socio-economic and environmental factors
Source: Sci Rep. 2019 Dec 11;9:18878. doi: 10.1038/s41598-019-55005-0 (PMC6906380; doi:10.1038/s41598-019-55005-0)
Supplement: Supplementary file 1 — Supplementary materials [file 41598_2019_55005_MOESM1_ESM.doc]

**Supplementary materials**

**Title of the article:** Pregnancy vulnerability in urban areas: a pragmatic approach combining behavioral, medico-obstetrical, socio- economic and environmental factors

**Author names**

Alice Brembilla, Nadine Bernard, Sophie Pujol, Anne-Laure Parmentier, Astrid Eckman, Anne-Sophie Mariet, Hélène Houot, Quentin Tenailleau, Gérard Thiriez, Didier Riethmuller, Marie Barba-Vasseur, Frédéric Mauny

**Supplementary tables**

**Supplementary table S1.** Number of vulnerability markers and percentages of adverse pregnancy outcomes (N=3686)

| Number of  vulnerabilities  among the 15  markers | Preterm birth | | Low birthweight | | Preeclampsia or  eclampsia | | Vaginal bleeding (2nd or 3th trimester) | | At least one adverse pregnancy outcome | |
| --- | --- | --- | --- | --- | --- | --- | --- | --- | --- | --- |
|  | N (%) | P | N (%) | P | N (%) | P | N (%) | P | N (%) | P |
| 0 (N=758) | 42 (5.5) | <0.001^a^ | 35 (4.6) | <0.001^a^ | 4 (0.5) | <0.001^a^ | 12 (1.6) | 0.002^a^ | 62 (8.2) | <0.001^a^ |
| 1 (N=1113) | 74 (6.6) | 0.008^b^ | 73 (6.6) | 0.001^b^ | 17 (1.5) | <0.001^b^ | 24 (2.2) | 0.013^b^ | 124 (11.1) | <0.001^b^ |
| 2 (N=884) | 60 (6.8) |  | 69 (7.8) |  | 31 (3.5) |  | 35 (4.0) |  | 123 (13.9) |  |
| 3 (N=555) | 51 (9.2) |  | 51 (9.2) |  | 20 (3.6) |  | 19 (3.4) |  | 94 (16.9) |  |
| ≥4 (N=376) | 40 (10.6) |  | 41 (10.9) |  | 31 (8.2) |  | 15 (4.0) |  | 81 (21.5) |  |
| Overall | 267 (7.2) |  | 269 (7.3) |  | 103 (2.8) |  | 105 (2.8) |  | 484 (13.1) |  |

^a^ Significance level of Cochran-Armitage trend test

^b^ Significance level of Chi-square test

**Supplementary table S2. Number of vulnerability dimensions and percentages of adverse pregnancy outcomes (N=3686)**

| Number of  vulnerability  dimensions among  the six | Preterm birth | | Low birthweight | | Preeclampsia or  eclampsia | | Vaginal bleeding (2nd or 3th trimester) | | At least one adverse pregnancy outcome | |
| --- | --- | --- | --- | --- | --- | --- | --- | --- | --- | --- |
|  | N (%) | P | N (%) | P | N (%) | P | N (%) | P | N (%) | P |
| 0 (N=758) | 42 (5.5) | 0.002^a^ | 35 (4.6) | <0.001^a^ | 4 (0.5) | <0.001^a^ | 12 (1.6) | 0.008^a^ | 62 (8.2) | <0.001^a^ |
| 1 (N=1250) | 82 (6.6) | 0.032^b^ | 82 (6.6) | 0.001^b^ | 24 (1.9) | <0.001^b^ | 30 (2.4) | 0.013^b^ | 145 (11.6) | <0.001^b^ |
| 2 (N=985) | 79 (8.0) |  | 82 (8.3) |  | 36 (3.7) |  | 37 (3.8) |  | 147 (14.9) |  |
| 3 (N=505) | 43 (8.5) |  | 50 (9.9) |  | 23 (4.6) |  | 22 (4.4) |  | 92 (18.2) |  |
| ≥4 (N=188) | 21 (11.2) |  | 20 (10.6) |  | 16 (8.5) |  | 4 (2.1) |  | 38 (20.2) |  |

^a^ Significance level of Cochran-Armitage trend test

^b^ Significance level of Chi-square test

**Supplementary table S3.** Sensitivity analysis: Vulnerability markers and odds-ratio of adverse pregnancy outcomes, omitting three markers

|  | At least one pregnancy outcome  OR (95% CI, p)  Omitting diabetes | At least one pregnancy outcome  Omitting genitourinary infection | At least one pregnancy outcome  Omitting HTA |
| --- | --- | --- | --- |
| Number of markers | P<0.001 | P<0.001 | P<0.001 |
| 0 | 1 | 1 | 1 |
| 1 | 1.43 (1.05-1.97) | 1.49 (1.10-2.01) | 1.17 (0.87-1.57) |
| 2 | 1.81 (1.31-2.49) | 2.08 (1.53-2.82) | 1.42 (1.05-1.92) |
| 3 | 2.49 (1.78-3.49) | 2.51 (1.79-3.52) | 1.78 (1.29-2.46) |
| ≥4 | 3.17 (2.20-4.55) | 3.37 (2.35—4.84) | 1.90 (1.32-2.72) |
|  |  |  |  |

**Supplementary table S4.** Sensitivity analysis: Vulnerability dimensions and odds-ratio of adverse pregnancy outcomes, omitting three markers

|  | At least one pregnancy outcome  OR (95% CI, P)  Omitting diabetes | At least one pregnancy outcome  Omitting genitourinary infection | At least one pregnancy outcome  Omitting HTA |
| --- | --- | --- | --- |
| Number of dimensions | P<0.001 | P<0.001 | P<0.001 |
| 0 | 1 | 1 | 1 |
| 1 | 1.49 (1.10-2.03) | 1.52 (1.14-2.04) | 1.21 (0.91-1.62) |
| 2 | 1.99 (1.46-2.72) | 2.26 (1.68-3.05) | 1.47 (1.09-1.97) |
| 3 | 2.58 (1.83-3.64) | 2.76 (1.96-3.89) | 1.92 (1.38-2.66) |
| ≥4 | 3.16 (2.02-4.92) | 3.17 (2.00-5.02) | 1.70 (1.07-2.72) |

**Supporting information**

| **Supporting information.** Description of the first selection of 39 socioeconomic variables at the IRIS scale | |
| --- | --- |
| **Variable group** | **Variable name and type** |
| Family and household | Proportion of people under the age of 25 in the total population (%) |
|  | Proportion of people over the age of 65 in the total population (%) |
|  | PROPORTION OF SINGLE-PARENT FAMILIES IN THE TOTAL POPULATION (%) |
|  | Proportion of householders living alone in the total population (%) |
| Immigration and mobility | PROPORTION OF FOREIGN PEOPLE IN THE TOTAL POPULATION (%) |
|  | PROPORTION OF FOREIGN IMMIGRANTS IN THE TOTAL POPULATION (%) |
|  | Proportion of people over 5 who lived in another municipality five years earlier (%) |
| Employment and income | *Proportion of people in the labor force in the 15-64 years old population (%)*^a^ |
|  | Proportion of men in the labor force in the 15-64 years old male population (%)^a^ |
|  | Proportion of women in the labor force in the 15-64 years old female population (%)^a^ |
|  | *PROPORTION OF UNEMPLOYED PEOPLE IN THE 15-64 YEARS OLD LABOR FORCE (%)*^b^ |
|  | Proportion of unemployed people in the male labor force (%)^b^ |
|  | Proportion of unemployed people in the female labor force (%)^b^ |
|  | Proportion of unemployed people in the 15-24 years old labor force (%) |
|  | PROPORTION OF UNEMPLOYED PEOPLE IN THE 55-64 YEARS OLD LABOR FORCE (%) |
|  | Proportion of self-employed (independent workers, employers…) in the labor force (%) |
|  | Proportion of farmers in the labor force (%) |
|  | PROPORTION OF MANAGER, CRAFTSPEOPLE AND SHOPKEEPERS IN THE LABOR FORCE (%) |
|  | PROPORTION OF BLUE-COLLAR WORKERS IN THE LABOR FORCE (%) |
|  | MEDIAN INCOME PER CONSUMPTION UNIT (IN EUROS PER YEAR) |
| Education | Proportion of people 6-14 years old attending school in the 6-14 years old population (%) |
|  | PROPORTION OF PEOPLE WITH NO SCHOOL GRADUATION (AND NOT STUDYING) IN THE 15 YEARS OLD AND MORE POPULATION (%) |
|  | Proportion of people with basic or intermediate general or vocation qualifications (and not studying) in the 15 years old and more population (%) |
|  | Proportion of people with general or vocational maturity certificates (and not studying) in the 15 years old and more population (%) |
|  | PROPORTION OF PEOPLE WITH AT LEAST A LOWER TERTIARY EDUCATION (AND NOT STUDYING) IN THE 15 YEARS OLD AND MORE POPULATION (%) |
|  | PROPORTION OF PEOPLE WITH A HIGHER EDUCATIONAL DEGREE (AND NOT STUDYING) IN THE 15 YEARS OLD AND MORE POPULATION (%) |
|  | Proportion of students in the 15 years old and more population (%) |
| Housing | PROPORTION OF INDIVIDUAL HOUSES IN THE MAIN RESIDENCES (%) |
|  | Proportion of multiple dwelling units in the main residences (%) |
|  | PROPORTION OF NON-OWNER-OCCUPIED IN THE MAIN RESIDENCES (%) |
|  | Proportion of main residences built before 1974 (%) |
|  | Proportion of main residences built after 1990 (%) |
|  | Proportion of main residences less than 40 m^2^ (%) |
|  | PROPORTION OF MAIN RESIDENCES LARGER THAN 100 M2 (%) |
|  | Proportion of main residences without bathtub or shower (%) |
|  | PROPORTION OF MAIN RESIDENCES WITH A PARKING SPACE (%) |
|  | AVERAGE NUMBER OF PEOPLE PER ROOM |
|  | PROPORTION OF HOUSEHOLDS WITHOUT A CAR (%) |
|  | PROPORTION OF HOUSEHOLDS WITH 2 OR MORE CARS (%) |

^a^ Redundant group “labor force”

^b^ Redundant group “unemployment ”

Italic: Variables selected during the “reduction of redundant groups” (step 1)

UPPERCASE: Variables selected during the step 2 of the procedure
